# Incidence and risk factors for chronic postsurgical pain following video-assisted thoracoscopic surgery: a retrospective study

**DOI:** 10.1186/s12893-022-01522-1

**Published:** 2022-03-02

**Authors:** Yingying Zhang, Rong Zhou, Bailing Hou, Suhong Tang, Jing Hao, Xiaoping Gu, Zhengliang Ma, Juan Zhang

**Affiliations:** Department of Anesthesiology, Nanjing Drum Tower Hospital, The Affiliated Hospital of Nanjing University Medical School, 321 Zhongshan Road, Nanjing, 210008 Jiangsu China

**Keywords:** Chronic postsurgical pain, Video-assisted thoracoscopic surgery, Predictors, Multivariate logistic regression

## Abstract

**Background:**

Video-assisted thoracoscopic surgery (VATS) has been widely used as an alternative for thoracotomy, but the reported incidence of chronic postsurgical pain (CPSP) following VATS varied widely. The purpose of this study was to investigate the incidence and risk factors for CPSP after VATS.

**Methods:**

We retrospectively collected preoperative demographic, anesthesiology, and surgical factors in a cohort of patients undergoing VATS between January 2018 and October 2020. Patients were interviewed via phone survey for pain intensity, and related medical treatment 3 months after VATS. Univariate and multivariate analysis were used to explore independent risk factors associated with CPSP.

**Results:**

2348 patients were included in our study. The incidence of CPSP after VATS were 43.99% (n = 1033 of 2348). Within those suffering CPSP, 14.71% (n = 152 of 1033) patients reported moderate or severe chronic pain. Only 15.23% (n = 23 of 152) patients with moderate to severe chronic pain sought active analgesic therapies. Age < 65 years (OR 1.278, 95% CI 1.057–1.546, *P* = 0.011), female (OR 1.597, 95% CI 1.344–1.898, *P* < 0.001), education level less than junior school (OR 1.295, 95% CI 1.090–1.538, *P* = 0.003), preoperative pain (OR 2.564, 95% CI 1.696–3.877, *P* < 0.001), consumption of rescue analgesia postoperative (OR 1.248, 95% CI 1.047–1.486, *P* = 0.013), consumption of sedative hypnotic postoperative (OR 2.035, 95% CI 1.159–3.574, *P* = 0.013), and history of postoperative wound infection (OR 5.949, 95% CI 3.153–11.223, *P* < 0.001) were independent risk factors for CPSP development.

**Conclusions:**

CPSP remains a challenge in clinic because half of patients may develop CPSP after VATS.

*Trial registration* Chinese Clinical Trial Registry (ChiCTR2100045765), 2021/04/24

## Introduction

Chronic postsurgical pain (CPSP) is defined as chronic pain that develops or increases in intensity after a surgical procedure or a tissue injury and persists beyond the healing process, i.e., at least 3 months after the surgery or tissue trauma by International Classification of Diseases-11 (ICD-11) [1], and its incidence varies from 3 to 85% according to surgery type [2]. As traumatic as thoracotomy, the reported incidence of CPSP can be up to 57% [3]. Since video-assisted thoracoscopic surgery (VATS) was introduced into clinical practice in the early 1990s, it has been widely used as an alternative to thoracotomy over the past 30 years [4]. Though it was considered less injury than thoracotomy and with relief of postoperative acute pain, the incidence of CPSP after VATS has been reported to range from 7.7 to 50% [1]. Limited by small sample size and inconsistent follow-up time, the reported incidence of CPSP following VATS varies widely and we can’t reach a comprehensive summary about the occurrence of CPSP after VATS at present.

The aetiology of CPSP after VATS is multifactorial and may involve both patient- and treatment-related factors. Although some studies have examined perioperative risk factors for the development of CPSP after VATS, most of them focused on a limited number of variables [5–7]. Since CPSP has been associated with long-term opioids use, unnecessary psychological pressure and reduced quality of life, identifying risk factors related to CPSP after VATS will be helpful for clinicians to carry out targeted prevention and help patients to form appropriate expectations [8].

Based on the above reasons, the primary aim of this study was to investigate the incidence of CPSP after VATS in a large sample of patients. The second aim was to identify independently predictors of CPSP from a comprehensive evaluation of demographic, anesthesiology, and surgical factors in a retrospective cohort.

## Methods

### Study design and population

This retrospective, observational study was approved by the Institutional Review Board (IRB) for Clinical Investigations at Nanjing Drum Tower Hospital, The Affiliated Hospital of Nanjing University Medical School (2020-297-02) and retrospective requirement for written informed consent was waived. This study was registered at Chinese Clinical Trial Registry (ChiCTR2100045765). Patients who underwent VATS between January 2018 and December 2020 at our institution (Nanjing Drum Tower Hospital, The Affiliated Hospital of Nanjing University Medical School) were identified. Study inclusion criteria were as follows: (1) age ≥ 18 years; (2) American Society of Anesthesiologists(ASA) I–III grade; (3) non-emergency VATS surgery. The exclusion criteria were as follows: (1) patients with previous thoracodorsal surgery; (2) patients underwent bilateral surgery or converted to thoracotomy; (3) patients who developed III/IV/V complications according to Clavien-Dindo grade system; (4) patients with inadequate medical records perioperatively.

### Data collection

Data on demographics, medical history, anesthesia/surgery related parameters, perioperative pain-related parameters were collected from an electronic medical records retrieval system.

Total intravenous anesthesia protocol was performed. No premedication was administered before surgery. Anesthesia was induced by intravenous midazolam 0.05 mg/kg, 1% propofol 1–2 mg/kg (or etomidate 0.3–0.5 mg/kg), fentanyl 3–8 μg/kg, vecuronium bromide 0.08–0.12 mg/kg. Double-lumen endotracheal intubation was performed under visual laryngoscope. Anesthesia was maintained with continuous infusion of propofol 4–12 mg/kg/h, remifentanil 0.05–0.2 μg/kg/min, cisatracurium 1–3 μg/kg/min. Intravenous patient-controlled analgesia consisted of a combination of sufentanil 1 µg/mL, ondansetron 8 mg, and dexmedetomidine 10 mg at a continuous infusion rate of 2 mL/h and a bolus of 0.5 mL with a lockout interval of 15 min. Based on the anesthesia record sheet, whether using nerve block, dexmedetomidine, and sevoflurane, dosage of fentanyl and remifentanil per kilogram, and intraoperative blood transfusion were collected.

VATS was performed by a two-port technique, one port was used as an operating hole and the other as an observation hole. The location of the chest wall incision was selected by surgeons according to the clinical features of patient’s lesion. At the end of surgery, the chest tube was disposed for postoperative thoracic drainage. The etiology of the operation, duration of the operation, whether lymph node dissection was performed and the amount of intraoperative blood loss were collected according to the surgery records.

If there were no contraindications, all patients received intravenous injection of propacetamol hydrochloride after operation routinely. If patients complained severe pain, surgeons choosed indomethacin, flurbiprofen axetil injection, codeine phosphate, pethidine or tramadol as rescue analgesics according to the patient’s pain severity. The use of rescue analgesics was extracted from the electronic medical system.

#### Evaluation of CPSP

Patients were contacted by telephone about whether CPSP developed after VATS. Our list of questions can be divided into two main parts:

Part 1. To confirm whether CPSP developed. The following questions were asked:

1. Did you feel any pain around the surgery scar or the surrounding issue 3 months after VATS?
2. Did you feel pain before surgery? Was the pain 3 months after VATS the same as the preoperative pain?
3. Was there any other cause for the pain (recurrence of malignant tumor or chronic wound infection)?

Part 2. To evaluate the intensity of CPSP and record the patients’ pain relief methods. The following questions were asked:

1. If 0 represents no pain and 10 represents the worst pain you can imagine, which number will reflects your most severe pain 3 months after VATS appropriately?
2. Did you adopt any measure to solve the pain, such as having a rest or reducing daily activity, taking medicine on yourself, asking help from doctors or none [9]?

If CPSP was confirmed by part 1, further understanding of the patient's CPSP characteristics could be obtained through part 2. Numerical Rating Scale (NRS) was used to evaluate patients’ pain intensity and the most severe pain could be induced when rest, coughing, moving or others. Pain score ≥ 1 were diagnosed as CPSP [10].

### Statistical analysis

Data were analyzed using SPSS 19.0 (SPSS, Inc., Chicago, IL, USA). The results were expressed as means ± standard deviation for continuous variables or as number and percentages for categorical variables. The independent t test or Mann–Whitney U-tests were used for between-group testing, depending on the distribution of the variables. Chi-square test tests or Fisher’s exact tests were applied for categorical variables.

Univariable logistic regression analysis was applied to examine predictors of CPSP after VATS, and candidate covariates were chosen based on statistical significance or possible clinical importance. Multivariate model was developed using a stepwise forward approach. The discriminatory power of the multivariate model was evaluated by using the area under the receiver operating characteristic curve and its 95% confidence interval (CI). The calibration of the multivariate model was evaluated using the Hosmer–Lemeshow goodness-of-fit statistic, where a high *P*-value indicates good calibration. A significance level of 5% (*P* < 0.05), and confidence intervals of 95% were used [11].

## Results

**Fig. 1:**
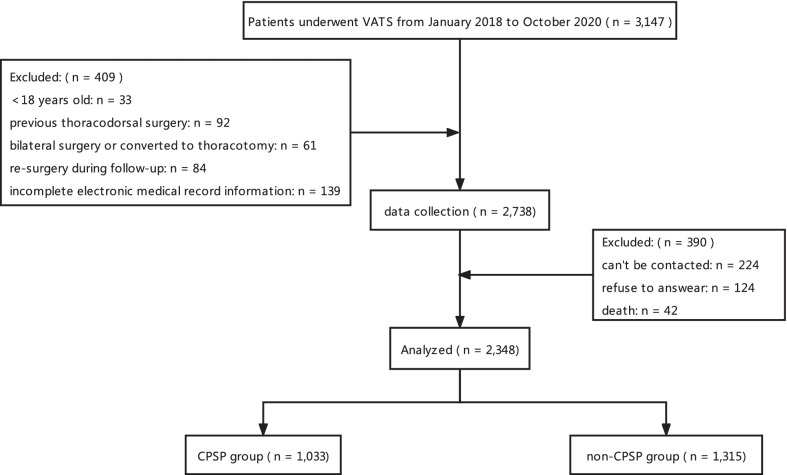
Flow diagram for patient inclusion

Of 3147 patients who were screened for inclusion, 2738 met the inclusion criteria. When we attempted to contact these patients and assess their postoperative pain, 348 patients were excluded due to unanswered phone calls/refusal to answer, and 42 were excluded due to death. Finally, 2348 patients were included in our analysis (Fig. 1). According to the CPSP definition from ICD-11, 43.99% (n = 1,033 of 2348) patients reported characteristics of CPSP after surgery with approximately 14.71% (n = 152 of 1033) reported moderate to severe pain (NRS > 3). Within those who reported moderate to severe pain, 22.52% (n = 34 of 152) did not take any treatment, 62.25% (n = 94 of 152) relieved their pain by resting or reducing activity, only 15.23% (n = 23 of 152) sought active analgesic therapies (medicine or consultation to doctors).

Sociodemographic and medical history of patients were presented in Table 1. Factors associated with CPSP included age, sex, smoking history, drinking history, education level, and preoperative pain (*P* < 0.05).

**Table 1:** Sociodemographic and medical history of subjects without and with CPSP (n = 2348)

Variables	Non-CPSP (n = 1315)	CPSP (n = 1033)	P value
Age, n (%)			
< 65	897 (68.2)	757 (73.3)	**0.008**
≥ 65	418 (31.8)	276 (26.7)	
Female, n (%)	627 (47.7)	623 (60.3)	** < 0.001**
BMI, n (%)			
< 24 kg/m^2^	722 (54.9)	605 (58.6)	0.076
≥ 24 kg/m^2^	593 (45.1)	428 (41.4)	
ASA, n (%)			
I/II	156 (11.9)	98 (9.5)	0.118
III	1159 (88.1)	931 (90.5)	
Smoking history, n (%)	200 (15.2)	121 (11.7)	**0.014**
Drinking history, n (%)	143 (10.9)	84 (8.1)	**0.026**
Hypertension, n (%)	358 (27.2)	273 (26.4)	0.666
Diabetes mellitus, n (%)	128 (9.7)	92 (8.9)	0.494
CHD, n (%)	36 (2.7)	20 (1.9)	0.206
Surgery history, n (%)	413 (31.4)	413 (31.2)	0.903
Preoperative pain, n (%)	36 (2.7)	75 (7.3)	**< 0.001**
Education level less than junior school, n (%)			
	658 (50.0)	591 (57.2)	**0.001**
Consumption of sedative hypnotic preoperative, n (%)			
	458 (34.8)	338 (32.7)	0.284

bold: *P*< 0.05

*BMI* body mass index, *ASA* American Society of Anesthesiologists, *CHD* coronary heart disease

Data about surgery and anesthesia were summarized in Table 2. Statistically significant differences were observed, including the consumption of fentanyl dose per kilogram (μg/kg), blood loss volume intraoperative (ml/kg), the consumption of rescue analgesia postoperative, the consumption of sedative hypnotic postoperative, and history of postoperative wound infection (*P* < 0.05).

**Table 2:** Surgery and anesthesia data of subjects without and with CPSP (n = 2348)

Variables	Non-CPSP (n = 1315)	CPSP (n = 1033)	*P* value
Anesthesia, n (%)			
General anesthesia	965 (73.4)	771 (74.6)	0.492
Combined with nerve block	350 (26.6)	262 (25.4)	
Fentanyl dosage (μg/kg)	11.3 ± 4.8	11.0 ± 4.3	**0.044**
Remifentanil dosage (μg/kg)	11.8 ± 10.1	12.1 ± 9.3	0.478
Dexmedetomidine usage, n (%)	1214 (92.3)	960 (92.9)	0.573
Sevoflurane usage, n (%)	221 (16.8)	199 (19.3)	0.123
PCIA, n (%)	955 (72.6)	763 (73.9)	0.501
Surgical Procedure, n (%)			
Lung	1235 (93.9)	967 (93.6)	0.179
Mediastinal	76 (5.8)	57 (5.5)	
Others	4 (0.3)	9 (0.9)	
Lymph node dissection, n (%)	603 (56.2)	470 (43.8)	0.863
Duration of surgery (min)	106.4 ± 47.6	107.9 ± 44.9	0.189
Blood loss (ml/kg)	2.0 ± 4.1	1.8 ± 3.5	**0.013**
Infusion volume (ml/kg)	22.7 ± 8.8	22.1 ± 8.0	0.114
Consumption of rescue analgesia postoperative, n (%)			
	450 (34.2)	411 (39.8)	**0.005**
Consumption of sedative hypnotic postoperative, n (%)			
	22 (1.7)	32 (3.1)	**0.022**
Subcutaneous emphysema postoperative, n (%)			
	210 (16.0)	185 (17.9)	0.212
History of postoperative wound infection, n (%)			
	12 (0.9)	57 (5.5)	**< 0.001**
Postoperative pulmonary infection, n (%)			
	31 (2.4)	37 (3.6)	0.079
Postoperative WBC (10^9^)	11.4 ± 3.1	11.3 ± 3.3	0.202
Postoperative CRP (mg/L)	52.9 ± 1.0	51.1 ± 1.1	0.205
PONV, n (%)	180 (13.7)	149 (14.4)	0.610

bold: *P*< 0.05

*PCIA* postoperative patient-controlled intravenous analgesia, *WBC* white blood cell, *CRP* C-reactive protein, *PONV* postoperative nausea and vomiting

**Fig. 2:**
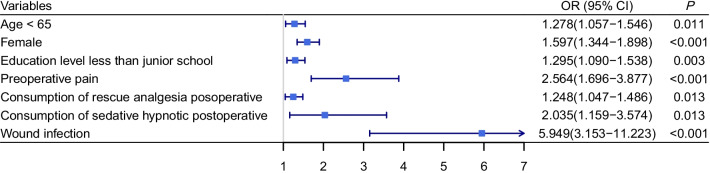
Multivariate model for CPSP after VATS

Based on statistical significance or possible clinical implication, variates with *P* < 0.25 in the univariate analysis were entered in the multivariate model. As showed in Fig. 2, seven risk factors were identified for CPSP after VATS: age < 65 years (OR 1.278, 95% CI 1.057–1.546, *P* = 0.011), female (OR 1.597, 95% CI 1.344–1.898, P < 0.001), education level less than junior school (OR 1.295, 95% CI 1.090–1.538, *P* = 0.003), preoperative pain (OR 2.564, 95% CI 1.696–3.877, *P* < 0.001), consumption of rescue analgesia postoperative (OR 1.248, 95% CI 1.047–1.486, *P* = 0.013), consumption of sedative hypnotic postoperative (OR 2.035, 95% CI 1.159–3.574, *P* = 0.013), and history of postoperative wound infection (OR 5.949, 95% CI 3.153–11.223, *P* < 0.001).

**Fig. 3:**
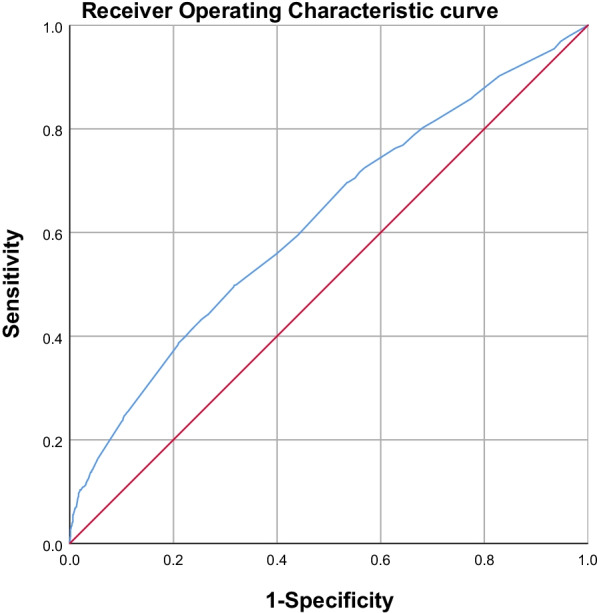
The area under the ROC curve of CPSP multivariate model

The predictive model for CPSP after VATS yield the area under the receiver operating characteristic curve of 0.622 (95% CI 0.599–0.644) (Fig. 3), and the model showed good calibration by Hosmer–Lemeshow goodness-of-fit statistic with *X*^*2*^ = 4.956, P = 0.665.

## Discussion

Although VATS alleviates some iatrogenic injuries, it is still controversial whether this technique can reduce the risk of CPSP. The reported rates of CPSP after VATS varies widely, such a difference between studies may be related to the methodology (uni-portal vs. multi-portal VATS), perioperative analgesia regimen and CPSP definition. Additionally, “risk factor” has been a hot spot in the field of CPSP research in recent 10 years [12]. Surgical procedure, age, physical health, mental health, preoperative pain in the surgical field and another area have been proved to be risk factors for the occurrence of CPSP after several surgical procedures [13, 14].

In this study, we investigated the incidence of CPSP after two-ports VATS in 2348 patients. Our study showed that the incidence of CPSP was 43.99%. Most CPSP were mild and bearable, and the incidence of moderate-severe CPSP were 14.71%. The results of multivariate logistic regression analysis showed that age < 65 years, female, education level less than junior school, preoperative pain, consumption of rescue analgesia postoperative, consumption of sedative hypnotic postoperative, and history of postoperative wound infection were risk factors of CPSP.

Previously published meta-analysis reported the incidence of CPSP after thoracotomy was 57%, but data about the risk of developing CPSP after VATS were not sufficient to summarize [3, 15]. The reported incidences of CPSP after VATS range from 7.7 to 50% [1], apparently, our result ranks at a higher level of it. The relatively higher incidence may be explained by the definition of CPSP we used. The postoperative CPSP we calculated included pain of any intensity. Although some researchers believe that pain > 3 points is clinically significant, study has confirmed that CPSP of any intensity will lead to a decline in postoperative quality of life [8]. From our perspective, taking into account pain of 1–2 points will prevent clinicians and patients from ignoring those bothering mild CPSP. In addition to the high prevalence of CPSP, we were surprised to find that the majority of patients deal with CPSP in a negative way, only 15.23% of patients with moderate to severe pain sought active analgesic therapies (medicine or consultation to doctors), most of the remaining patients got relief from CPSP by resting or reducing activity. Overall, our results showed that although VATS significantly reduced the iatrogenic injury caused by surgical procedures, the incidence of CPSP after VATS remained high and most patients had a negative attitude towards CPSP management.

In terms of demographics data, age < 65 years, female and education level less than junior school were independent risk factors for CPSP. These results were consistent with previously published, known risk factors for CPSP in a variety of surgical procedures, including thoracic surgery [13, 14, 16].The relationship between age and CPSP can be explained by two points. First, young patients are biologically more sensitive to low-intensity noxious stimuli and may having a more heightened central nervous system responsiveness [17]. Second, from a physiological perspective, older adults are more conservative in pain perception and reporting and are more reluctant to report pain when it dose occurs [18]. The association between sex and CPSP can also be explained from these two aspects. From biologically, differences of sex hormone levels, pain-related receptor activity such as *N*-methyl-*d*-aspartic acid receptor or *P*2*X*3 receptor, μ/κ subtype splits in the endogenous analgesic system, and brain structure and function between men and women are related to the mechanism of sex differences in pain perception [8]. And from psychologically, female are more self-conscious and more likely to report pain to others [19]. Association between education level and CPSP has also been proved in several studies, but the specific mechanism is still unclear, pain catastrophizing may be a mediating factor between them [20]. Anyway, the combination of these demographic factors suggests a higher risk of developing CPSP. Recognition of these predictors, although unmodifiable, can help clinicians identify high-risk groups during preoperative evaluation and tailor an individualized pain treatment regimen [21].

As for perioperative pain-related parameters, our findings revealed that preoperative pain, the consumption of rescue analgesics and sedative hypnotic after surgery were independent predictors for CPSP. The association between preoperative pain and CPSP has been reported in several clinical trials [3, 13, 22]. The mechanism between preoperative pain and CPSP remains elusive but cumulative evidence has demonstrated that sensitization of the peripheral and central nervous system, which related to the alterations of peripheral nociceptors sensitivity and function of the pain descending inhibitory system may be the possible explanations between them [23]. Since preoperative pain could explain part of the interindividual variance in pain sensitivity, it may sensitize patients to new painful stimuli [24].

Postoperative use of rescue analgesics is an important indicator of postoperative pain intensity, especially when pain scores are performed only once or twice a day, the need for rescue analgesics helps in revealing the true level of postoperative pain intensity [25]. Consistent with the thoracic surgery and other postsurgical chronic pain conditions, we reported that postoperative consumption of rescue analgesics and sedative hypnotic in hospital (which indicates a higher severity of acute pain) was associated with a greater risk of developing CPSP [26]. Acute postoperative pain represents actual or potential tissue injury and motivates a response that removes the organism from such noxious stimuli [27]. The more severe the postoperative acute pain, the more severe the tissue injury, and the less adequate the pain control, which may induce peripheral sensitization and neuroplastic changes that involves altered pain processing [28]. An interesting study reported that although acute pain after thoracic surgery was comprised of thoracic pain, shoulder pain, and referred pain, only thoracic pain was closely related to the occurrence of CPSP. This study further highlights the importance of effective management of postoperative acute pain [29]. Although our results did not show that nerve block reduces the risk of CPSP, the latest meta-analysis did report a meaningful change in the incidence of CPSP by controlling postoperative acute pain through regional anesthesia [30]. Discrepancy between results may be related to differences in nerve block technique, and the type of study design. Anyway, the attempts to better manage postoperative acute pain are of great clinical significance in CPSP prevention.

For surgery related parameters, the history of postoperative wound infection was strongly associated with the occurrence of CPSP. On the one hand, nociceptor neurons themselves can detect pathogens and their related molecular ligands to mediate pain, on the other hand, immune cells secrete multiple cytokines during infection which can lower the threshold of action potentials of nociceptor [31]. In addition, cytokines and chemokines produced by activation of spinal astrocytes and microglias have been shown to be associated with chronic pain after infection, although the model used in this study was parasitic infection [32]. Peripheral and central sensitization due to infection may be responsible for the higher risk of CPSP. Our results showed no statistically significant difference in the contents of white blood cells, lymphocytes, neutrophils and CRP between the CPSP group and the non-CPSP group after surgery as Wang et al. [33], but this may be related to the fact that we only routinely rechecked relevant biochemical indicators on the first day postoperative. After discharge, some patients would suffer from out-of-hospital wound infection due to improper nursing or pleural effusion. However, due to the defects of retrospective study, we could not obtain relevant data. Although current studies have found a strong association between infection and acute pain, the specific mechanisms between infection and chronic pain need a further investigation.

Guidelines recommend intravenous use of dexmedetomidine during VATS and a number of studies have found that intraoperative use of dexmedetomidine can reduce pain within 24 h after VATS without significant adverse events [34], however the relationship between intraoperative use of dexmedetomidine and CPSP after VATS has not been specifically discussed. Although we didn’t find positive association between continuous pumping of dexmedetomidine with improvement in postoperative CPSP, it is worth noting that, as a routine medication, dexmedetomidine was used in about 92% of patients in our study population, which may have an impact on our results. A randomized controlled trial is needed to explore whether dexmedetomidine can lower the risk of CPSP after VATS.

Our study has strengths, including the large sample size and adequate range of risk factors collection. However, the results should still be interpreted cautiously for some reasons, mostly related to its retrospective design and the telephone interview. To solve these, We made a data acquisition table in advance to collect comprehensive and standardized data before, during and after surgery for each patient. During the telephone interview, we assessed patients’ CPSP according to a pre-formulated list of questions, which ensured the homogeneity of the follow-up process. Besides, due to the long follow-up time of CPSP, other authors often use telephone follow-up to obtain relevant data [8, 35]. Additionally, for the data collected from in a single institution, the results may be influenced by the selection bias.

## Conclusion

Overall, our study found that the incidence of CPSP after VATS was 43.99%. Although the majority of patients reported mild pain, such a high incidence suggests that CPSP after VATS remains an important challenge that cannot be ignored. In addition, our study found that younger age, female, low education level, preoperative pain, postoperative consumption of rescue analgesics and sedative hypnotic and postoperative wound infection were important predictors of CPSP, which suggested that clinicians should conducted a throughout evaluation perioperatively in order to identify high-risk groups of CPSP as soon as possible and tailor individualized pain prevention and treatment strategies for them.

## Data Availability

The datasets generated during and/or analyzed during the current study are available from the corresponding author on reasonable request.

## Abbreviations

VATS: Video-assisted thoracoscopic surgery
CPSP: Chronic postsurgical pain
CI: Confidence interval
OR: Odds ratio
ICD-11: International Classification of Diseases-11
IRB: The Institutional Review Board
ASA: American Society of Anesthesiologists
NRS: Numerical Rating Scale
BMI: Body mass index
CHD: Coronary heart disease
PCIA: Postoperative patient-controlled intravenous analgesia
WBC: White blood cell
CRP: C-reactive protein
PONV: Postoperative nausea and vomiting
